# Frequency pushing enhanced by an exceptional point in an atom–cavity coupled system

**DOI:** 10.1038/s41598-024-54008-w

**Published:** 2024-02-12

**Authors:** Joohye Lee, Jinuk Kim, Kyungwon An

**Affiliations:** 1 Department of Physics and Astronomy and Institute of Applied Physics, Seoul National University, Seoul, 08826 Korea; 2 Department of Physics, Yale University, New Haven, CT 06520 USA

**Keywords:** Quantum optics, Atomic and molecular interactions with photons

## Abstract

We observed the frequency pushing of the cavity resonance as a result of the coupling of the cavity field with the ground state ^138^Ba in a high-Q cavity. A weak probe laser propagated along the axis of a Fabry–Pérot cavity while ground-state barium atoms traversed the cavity mode perpendicularly. By operating the atom–cavity composite in the vicinity of an exceptional point, we could observe a greatly enhanced frequency shift of the cavity transmission peak, which was pushed away from the atomic resonance, resulting in up to 41 ± 7 kHz frequency shift per atom from the empty cavity resonance. We analyzed our results by using the Maxwell–Schrödinger equation and obtained good agreement with the measurements.

Microscopic lasers utilizing the strong interactions between atoms and a cavity field can serve as a test ground for various quantum optical phenomena such as nonclassical photon statistics^1–5^ and ultralow-threshold lasing^6–8^. In particular, an arrangement of atoms in a beam traversing a cavity in a short interaction time has advantages of preparing specific atomic states, fully inverted^9^ or in a superposition state^10^, and achieving steady-state operation while avoiding saturation effects. Due to the strong coupling between the atom and the cavity field^11–13^, the cavity resonance frequency can shift from its empty-cavity value when the cavity is detuned from the atomic resonance. Quantum frequency pulling phenomenon^14^ up to 2.1 kHz/per atom was observed in the cavity-QED microlaser as a result of the strong atom–cavity coupling for initially inverted atoms. In the recent coherent superradiance experiment^10,15,16^, the cavity frequency should be locked while the atoms in a quantum superposition of the ground and excited states interact with the cavity field. The sign and the amount of cavity resonance shift depends on the superposition states and the cavity locking frequency should be adjusted accordingly. Understanding the frequency shift of the cavity field is thus crucial, particularly in the cavity QED experiments employing superposition-state atoms.

The frequency shift of the cavity field occurs in opposite directions depending on the atomic state. For example, the frequency of the cavity field is pulled towards the atomic resonance when the atoms are mostly in the excited state. On the other hand, the frequency of the cavity field tends to be pushed away from the atomic resonance when the atoms are predominantly in the ground state. The former is called frequency pulling whereas the latter is frequency pushing.

Although frequency pulling has been widely studied in lasers, including some cases performed with a single ion in a cavity^17^, there have been limited studies on frequency pushing studies. Most of the frequency pushing measurements were done in free space^18–20^. Only one study was carried out with a vapor cell placed in a cavity^21^. To the best of our knowledge, there is no frequency-pushing experiment performed in a cavity-QED setting associated with strong as well as intermediate coupling regimes.

In this paper, we report the frequency pushing of the cavity resonance measured around the atomic resonance as a function of the cavity–atom detuning in an atom–cavity system where $^{138}$Ba atoms initially prepared in the ground state traverse the cavity mode continuously. The atom–cavity system for our measurements corresponds to near exceptional point condition in the language of non-Hermitian physics^22^. As we increase the atom–cavity coupling constant from the weak coupling regime toward the strong coupling regime, the frequency shift would increase. However, we can do so up to the exceptional point (EP), where two eigenstates coalesce to one so the cavity transmission still exhibits a single peak. Therefore, by choosing the near EP condition, we could enhance the frequency pushing up to $41\pm 7$ kHz, about 20 times larger than the largest frequency shift per atom observed in the cavity-QED microlaser^14^ while maintaining a single-peak cavity transmission lineshape. Moreover, the atomic dissipation via the absorption of the probe laser was maximized near the EP with zero atom–cavity detuning, as confirmed in the greatly increased linewidth as well as the reduced magnitude of the observed cavity transmission as a function of the probe-cavity detuning. We analyzed our measurements by using the Maxwell–Schrödinger equation and obtained good agreement between theory and experiment.

## Results

### Semiclassical description of frequency pushing

The expression for the probe transmission of the atom–cavity system can be obtained by using the semiclassical Maxwell–Schrödinger equations. The atoms initially unexcited are continuously injected into the cavity while a probe laser is coupled to the cavity mode. Under the slowly varying envelope approximation, the system is described by the following equations for the electric field $\mathcal {E}$ and the polarization $\mathcal {P}$—both slowly varying envelopes—as (see Methods for derivation)

1 $$ {\dot{\mathcal{E}}}(t) + (\gamma _{c}  - i\Delta _{c} ){\mathcal{E}}(t) = 2i\pi \omega _{P} {\mathcal{P}}\left( t \right) + i\frac{{\xi E_{0} }}{{2\omega }} $$

2 $$\begin{aligned} \dot{\mathcal {P}}(t)+(\gamma _p-i\Delta _{p})\mathcal {P}(t)= & {} i\frac{\mu ^2 N}{\hbar V}\mathcal {E}(t), \end{aligned}$$

where $\gamma _p$($\gamma _c$) is the damping rate (HWHM) of the polarization(cavity field), $\Delta _{p(c)}=\omega -\omega _{p(c)}$ with $\omega$ the probe frequency and $\omega _{p(c)}$ the resonance frequency of the atom(cavity), $E_0$ is the probe field amplitude, $\xi$ is the probe-cavity coupling (in the unit of frequency squared), $\mu$ is the induced dipole moment, *N* is the mean number of atoms in the cavity and *V* is the cavity mode volume.

We are interested in the steady-state solution with the atoms stationary in the cavity. The steady-state solution in this case is obtained by letting $\dot{\mathcal {P}}=0=\dot{\mathcal {E}}$. The resulting $\mathcal {E}$ is

3 $$\begin{aligned} \mathcal {E}=i\frac{\xi E_0}{2\omega }\frac{(\gamma _p-i\Delta _p)}{Ng^2+(\gamma _c-i\Delta _c)(\gamma _p-i\Delta _p)}, \end{aligned}$$

where $g\equiv \frac{|\mu |}{\hbar }\sqrt{\frac{2\pi \hbar \omega _p}{V}}$ is the atom–cavity coupling constant. Cavity transmission *T* is then proportional to $|\mathcal {E}|^2$

4 $$\begin{aligned} T\propto \Bigg |\frac{(\gamma _p-i\Delta _p)}{Ng^2+(\gamma _c-i\Delta _c)(\gamma _p-i\Delta _p)}\Bigg |^2. \end{aligned}$$

Equation (4), giving the cavity-transmission lineshape as a function of the probe laser frequency, is derived for stationary atoms in the cavity. In this case, the damping rate $\gamma _p$ of the induced dipole moment *p* or the decay rate of the off-diagonal element $\rho _{ab}$ of the density matrix equals half of the total radiative decay rate $\Gamma _0$ of level a to level b as well as to other metastable states (e.g. $^3$D$_{1,2}$ states for $^{138}$Ba atoms). If atoms are traversing the cavity mode at speed *v* perpendicularly to the cavity axis as in the experiment to be discussed below, the damping rate $\gamma _p$ should be modified in order to incorporate the transit time broadening. The induced dipole moment lasts in the cavity for a time duration equal to the transit time $\tau$ of each atom. This finite interaction time results in an extra dephasing of the induced dipole moment at a rate inversely proportional to the transit time^23^ in such a way that $\gamma _p$ is modified to

5 $$\begin{aligned} \gamma _p=\Gamma _0/2+\alpha /\tau , \end{aligned}$$

where $\alpha$ is a constant of the order of unity. The value of $\alpha$ can be determined by fitting the observed frequency pushing data with our model. The transit time $\tau$ is calculated by equating the pulse area experienced by the atom across the cavity mode of a Gaussian profile with mode waist $w_m$ to that of a flat top mode.

6 $$\begin{aligned} \tau =\int _{-\infty }^{\infty } e^{-(vt/w_m)^2} dt=\sqrt{\pi }\frac{w_m}{v}. \end{aligned}$$

The cavity transmission frequency $\omega _t$ for a given empty-cavity–atom detuning $\Delta _{cp}=\omega _c-\omega _p$ is given by the frequency corresponding to the maximum of the single-peak cavity transmission curve $T(\omega )$: $\left. \frac{\partial T(\omega )}{\partial \omega }\right| _{\omega =\omega _t}=0$. The amount of frequency pushing is then given by $\delta \omega =\omega _t-\omega _c$.

### Variation of the transmission lineshape with the dephasing rate

The cavity transmission lineshape given by Eq. (4) can be factorized into two Lorentzians and one inverted Lorentzian as follows.

**Figure 1:**
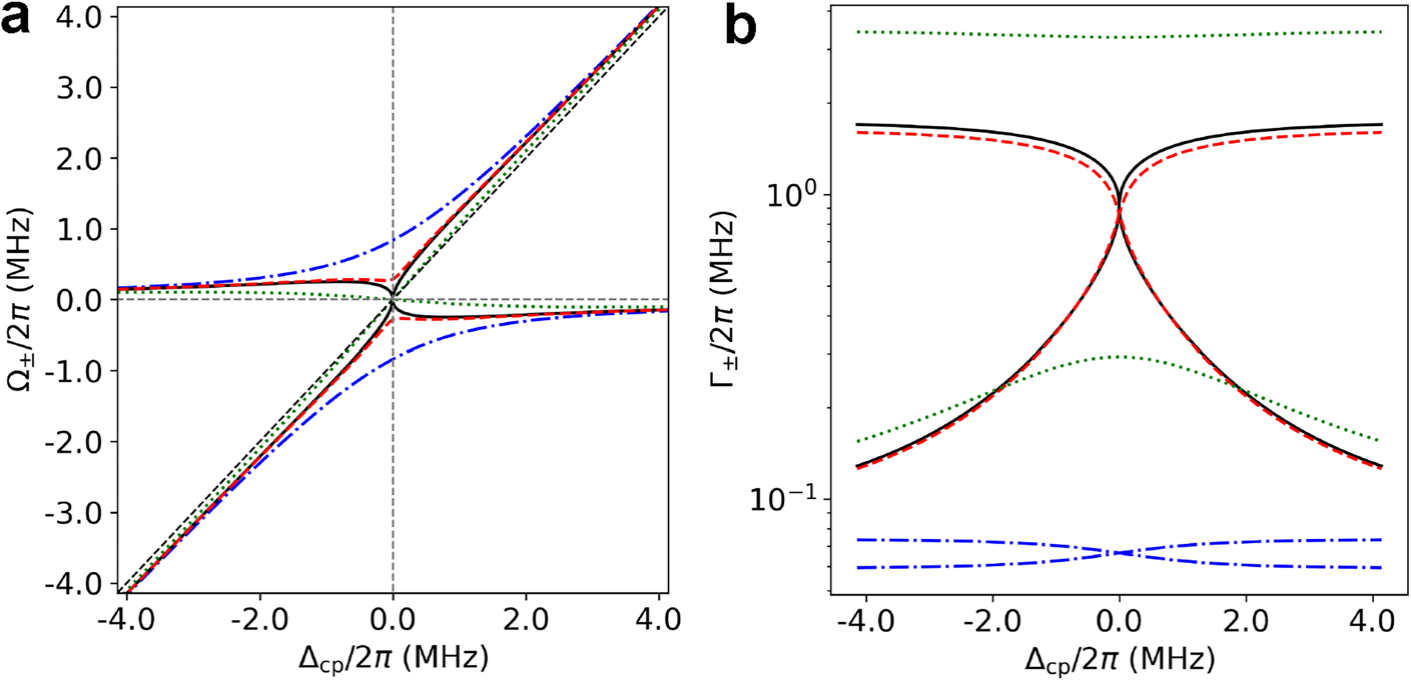
Eigenvalues of the atom–cavity system under various coupling conditions. (**a**) The real part $\Omega _\pm$ of the eigenvalues. (**b**) The imaginary part $\Gamma _\pm$ of the eigenvalues in log scale. Parameters in the actual experiments are used: $g/2\pi =348$ kHz, $\gamma _c/2\pi =74$ kHz and $N=5.8$. The damping rate $\gamma _p$ of the induced dipole moment is varied to simulate a strong coupling with $\gamma _p/2\pi =58.9$ kHz (dash-dot, blue), an EP with $\gamma _p/2\pi =1.750$ MHz (solid, black) and a weak coupling with $\gamma _p/2\pi =3.5$ MHz (dot, green). The intermediate coupling corresponding to the experiment is simulated with $\gamma _p/2\pi =1.65$ MHz (dashed, red), which was determined with $\tau =120$ ns and $\alpha =1.20$ in Eq. (5). The black dashed diagonal line that passes through the origin indicates $\omega =\omega _c$.

7 $$\begin{aligned} T(\omega )\propto \frac{\Delta _p^2+\gamma _p^2}{\left[ (\omega -\Omega _+)^2+\Gamma _+^2\right] \left[ (\omega -\Omega _-)^2+\Gamma _-^2\right] }. \end{aligned}$$

where $\Omega _\pm$ and $\Gamma _\pm$ are given by

8 $$\begin{aligned} \Omega _{\pm }=\omega _+\pm R, \;\;\; \Gamma _{\pm }=\gamma _+\pm I \end{aligned}$$

with

9 $$\begin{aligned} \ 2R^2= & {} (Ng^2+\omega _-^2-\gamma _-^2)+\sqrt{(Ng^2+\omega _-^2-\gamma _-^2)^2+4\omega _-^2\gamma _-^2}, \end{aligned}$$

10 $$\begin{aligned} 2I^2= & {} -(Ng^2+\omega _-^2-\gamma _-^2)+\sqrt{(Ng^2+\omega _-^2-\gamma _-^2)^2+4\omega _-^2\gamma _-^2}, \end{aligned}$$

11 $$\begin{aligned} \omega _{\pm }= & {} \frac{(\omega _c\pm \omega _p)}{2},~\gamma _{\pm }=\frac{(\gamma _c\pm \gamma _p)}{2}. \end{aligned}$$

In obtaining Eq. (7), the denominator in Eq. (4) is factorized with respect to $\omega$ as

12 $$\begin{aligned} |Ng^2+[\gamma _c-i(\omega -\omega _c)][\gamma _p-i(\omega -\omega _p)]|^2=|(\omega -\Omega _++i\Gamma _+)(\omega -\Omega _-+i\Gamma _-)|^2, \end{aligned}$$

which suggests that the atom–cavity system has two new eigenmodes, plus and minus modes, with eigenvalues $\lambda _\pm =\Omega _\pm -i\Gamma _\pm$, which are revealed in the cavity transmission lineshape.

We can obtain the same eigenvalues by considering the atom–cavity system described by the following non-Hermitian Hamiltonian^22,24^.

13 $$\begin{aligned} H=\hbar \begin{bmatrix} \omega _{\text{p}} -i\gamma _p &{} \sqrt{N}g \\ \sqrt{N}g &{} \omega _{\text{c}}-i\gamma _c \\ \end{bmatrix}, \end{aligned}$$

which is expressed in the single-quantum atom-field bases $|\text{e},0\rangle$ and $|\text{g},1\rangle$, where e(g) stands for atomic excited(ground) states and 0 and 1 indicate the number of photons in the cavity. The diagonal terms describe an atom(cavity) oscillator with resonance frequency $\omega _{p(c)}$ and a damping rate $\gamma _{p(c)}$ and the off-diagonal term indicates the coupling between two oscillators. We can obtain new eigenvalues by solving the secular equation

14 $$\begin{aligned} \begin{vmatrix} \omega _{\text{p}} -i\gamma _p -\lambda&\sqrt{N}g \\ \sqrt{N}g&\omega _{\text{c}}-i\gamma _c -\lambda \\ \end{vmatrix}=0 \end{aligned}$$

or

15 $$\begin{aligned} Ng^2+[\gamma _c-i(\lambda -\omega _c)][\gamma _p-i(\lambda -\omega _p)]=0. \end{aligned}$$

Comparing this with Eq. (12), we immediately recognize the new eigenvalues $\lambda _\pm =\Omega _\pm -i\Gamma _\pm$, respectively.

The variation of the eigenfrequencies $\Omega _\pm$ as a function of the cavity–atom detuning is depicted in Fig. 1 under various coupling conditions, that is, the strong ($\sqrt{N}g\gg |\gamma _-|$), the intermediate ($\sqrt{N}g\sim |\gamma _-|$ or near an exceptional point), and the weak coupling ($\sqrt{N}g\ll |\gamma _-|$) regimes. An exceptional point (EP) is where two eigenstates of a non-Hermitian system coalesce into a single eigenstate in the parameter space^22,24–27^. An EP occurs in the atom–cavity system when $\omega _c=\omega _p$ and $\sqrt{N}g=|\gamma _-|$. We can easily confirm that under the EP condition $R=I=0$ resulting in $\lambda _+=\lambda _-$. As indicated in Fig. 1, the frequency pushing measurements to be discussed below occurs in the intermediate coupling regime or near the exceptional point with the transit time broadening taken into account in $\gamma _p$ with $\tau =120$ ns and $\alpha =1.20$. For large positive or negative cavity–atom detuning, two eigenmodes appear as the cavity-like mode or atom-like mode depending on the sign of the atom–cavity detuning with their eigenfrequencies approaching $\omega _c$ or $\omega _p$ as shown in Fig. 1.

**Figure 2:**
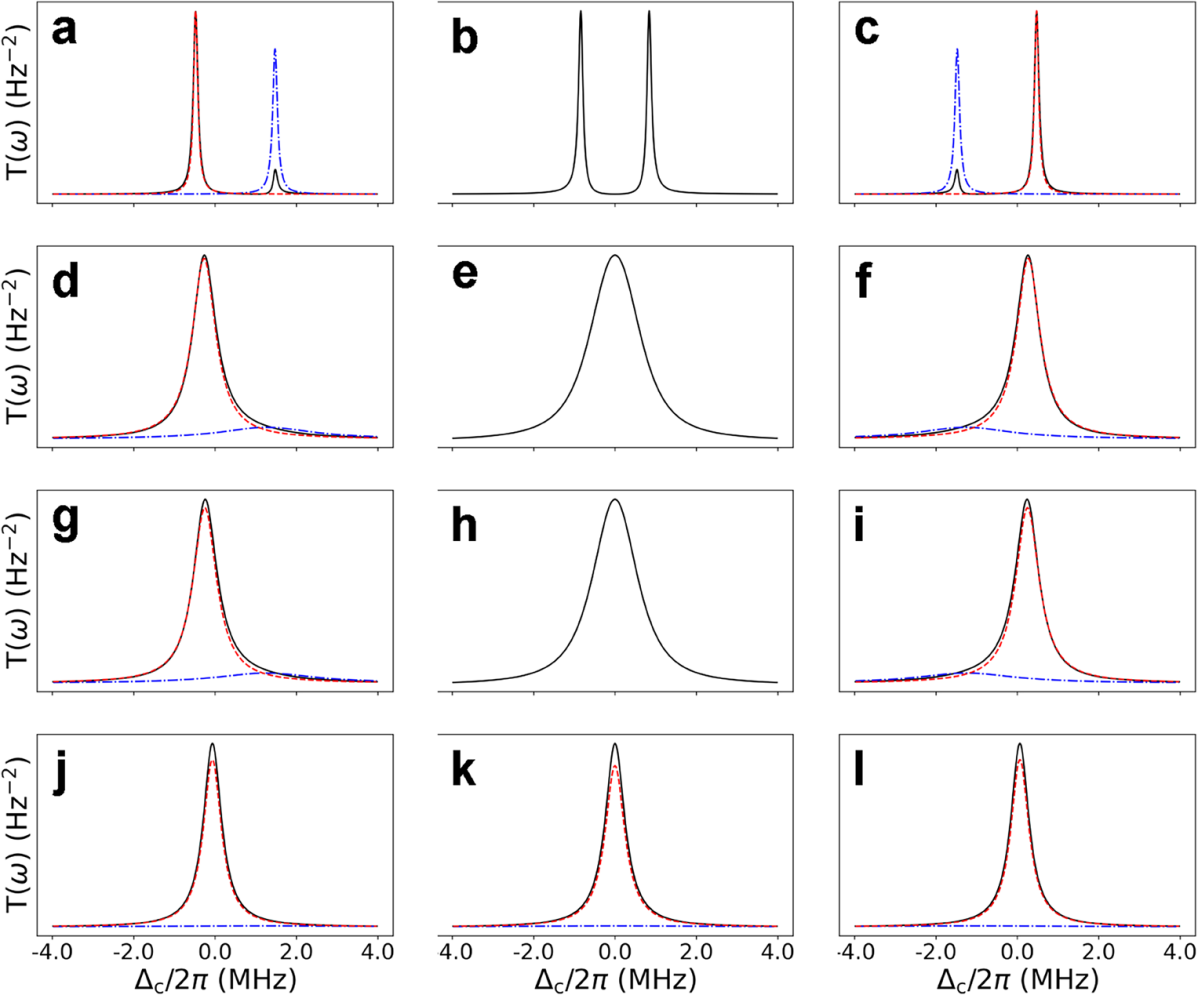
Cavity transmission lineshapes under the various coupling conditions. (**a**, **b**, **c**) for the strong coupling with $\gamma _p/2\pi =58.9$ kHz, (**d**, **e**, **f**) for the actual experiment with $\gamma _p/2\pi =1.65$ MHz, (**g**, **h**, **i**) for the EP with $\gamma _p/2\pi =1.750$ MHz and (**j**, **k**, **l**) for the weak coupling with $\gamma _p/2\pi =3.5$ MHz. The atom–cavity coupling constant is set to $g/2\pi =348$ kHz. Transmission line shapes (black solid curves) are shown along with the cavity-like lineshape (red dashed curve) and the atom-like lineshape (blue dash-dot curve) whenever relevant. The left, center, and right columns correspond to the cavity–atom detuning of − 1, 0, and + 1 MHz, respectively. The experiment near the EP is still toward the strong coupling, so we cannot distinguish atom-like and cavity-like modes near zero detuning in (**e**), and so do we in the strong coupling regime in (**b**) as well as at EP in (**h**).

Cavity transmission lineshapes under the various coupling conditions (corresponding to different rows) are shown in Fig. 2 for three representative cavity–atom detuning values of − 1 MHz, 0, and + 1 MHz (corresponding to the left, center, and right columns). In the strong coupling regime (the top row), the two eigenmodes are split approximately by $2\sqrt{N}g$. The Lorentzian line shapes $\mathcal {L}_\pm (\omega )$ corresponding to the plus and minus eigenmodes are defined as $\mathcal {L}_\pm (\omega )=1/(\Delta _\pm ^2+\Gamma _\pm ^2)$, respectively, with $\Delta _{\pm }=\omega -\Omega _{\pm }$. In the intermediate and weak coupling regimes, $\mathcal {L}_+ (\mathcal {L}_-)$ corresponds to the cavity(atom)-like mode for $\Delta _{cp}>0$ and atom(cavity)-like mode for $\Delta _{cp}<0$ with a choice of $R>0$. The sign of *I* is chosen as the same as the sign of $\gamma _-$ for $\Delta _{cp}>0$ and as the opposite of $\gamma _-$ for $\Delta _{cp}<0$ in order to ensure that the linewidth of the cavity(atom)-like mode is continuously transformed to $\gamma _c$($\gamma _p$) as $|\Delta _{cp}|\rightarrow \infty$ in these regimes.

### Experimental results

**Figure 3:**
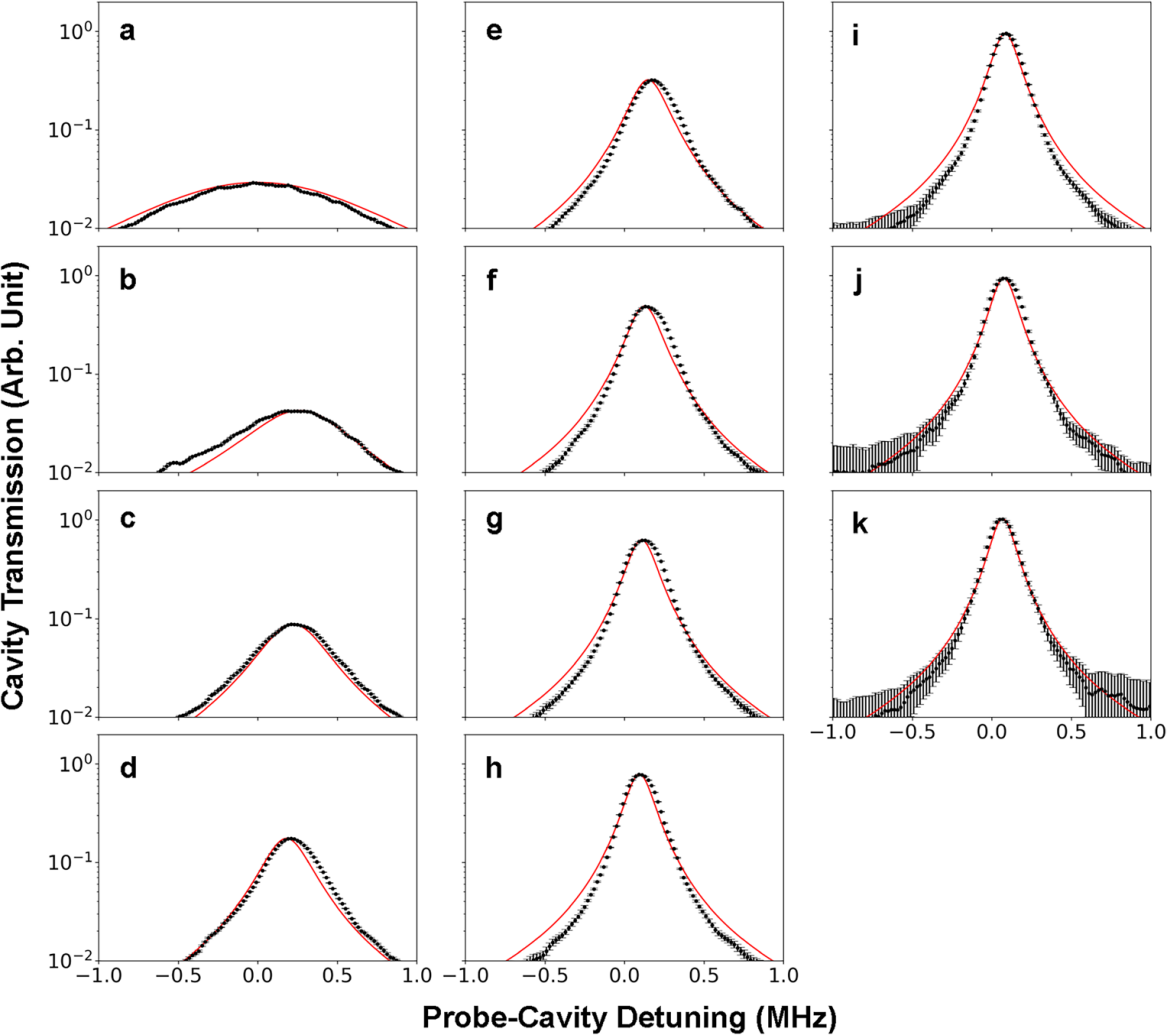
Observed cavity transmission lineshapes for various cavity–atom detunings. The lineshapes (dots) shown in (**a**–**k**) are measured for fixed cavity–atom detunings chosen from 0 [for (a)] to 10 MHz [for (k)] at a 1 MHz interval. The red curves are the theoretical curves given by Eqs. (4) and (7) with only one fitting parameter $\alpha =1.20$. The other parameters are either known or predetermined as $\gamma _c/2\pi =74$ kHz, $\Gamma _0/2\pi =117.8$ kHz, $g/2\pi =348$ kHz, $N=5.8$ and $\tau =120$ ns. The error bars in (**i**), (**j**) and (**k**) are relatively large because the probe power was low compared to the others.

Some of the observed cavity transmission lineshapes are shown in Fig. 3. Each lineshape is an average of about 20 repeated measurements. The peak positions represent the cavity resonances $\omega _t$ shifted due to the atom–cavity interaction.

**Figure 4:**
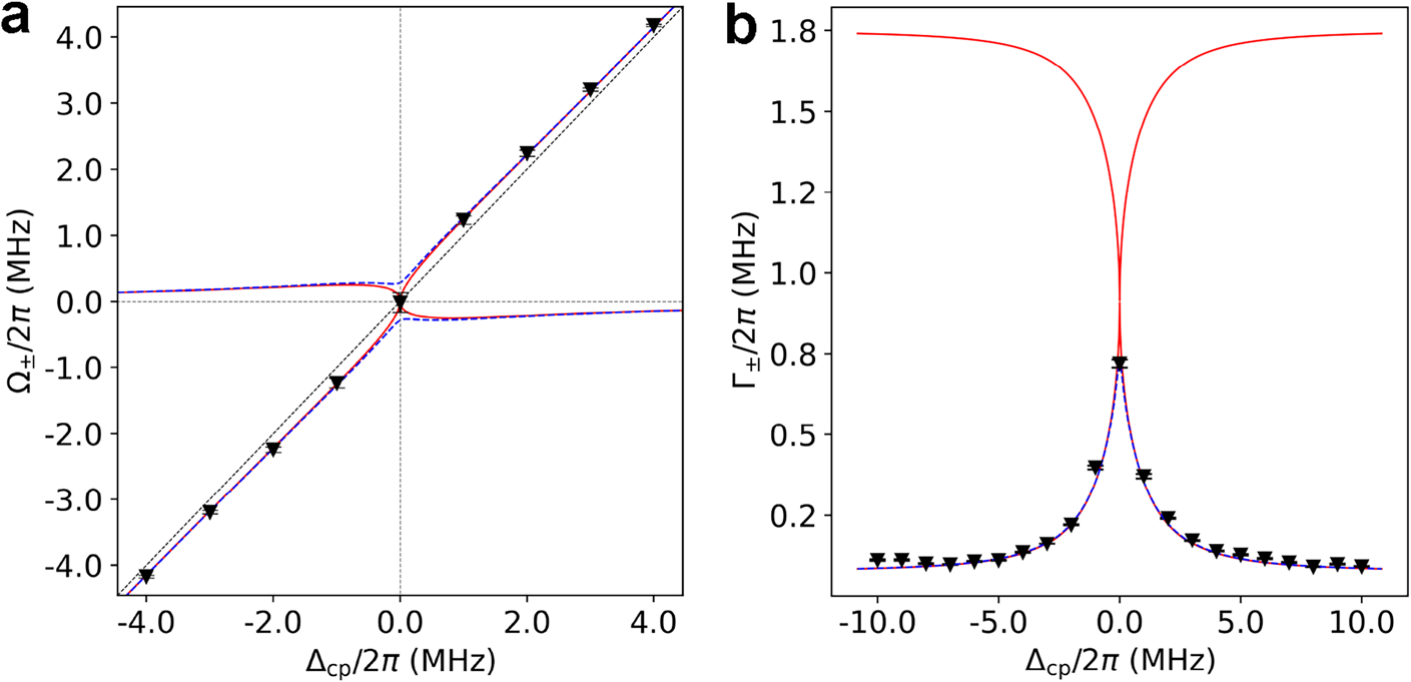
Observed real and imaginary parts of the eigenvalues for various cavity–atom detuning. (**a**) The real part $\Omega _\pm (\Delta _{cp})$ and (**b**) the imaginary part $\Gamma _\pm (\Delta _{cp})$ of the eigenvalues obtained from the cavity transmission lineshapes of Fig. 3 by fitting the lineshapes with Eq. (7). For the case of $\Delta _{cp}=0$, the signal-to-noise ratio was not enough to produce reliable $\Omega _\pm$ values, so the zero shift of the peak position is plotted instead. The red solid curve shows the $\Omega _\pm (\Delta _{cp})$ and $\Gamma _\pm (\Delta _{cp})$ for the EP condition for reference. Experimental results are shown as black inverted triangles and are fitted by the blue dashed curve corresponding to $\gamma _p/2\pi =1.65$ MHz.

By fitting the observed lineshapes with Eq. (7), we can obtain the real and imaginary parts of eigenvalues, and the results are summarized in Fig. 4 as black inverted triangles. The blue dashed curves in (a) and (b) of Fig. 4 represent the fit for the data with $\gamma _p/2\pi =1.65$ MHz whereas the red solid curves show the $\Omega _ \pm (\Delta _{cp})$ and $\Gamma _\pm (\Delta _{cp})$ for the EP condition for comparison. The experimental eigenvalue curves are practically indistinguishable from those corresponding to the EP except for the origin, indicating the high proximity of our experiment to the EP condition.

**Figure 5:**
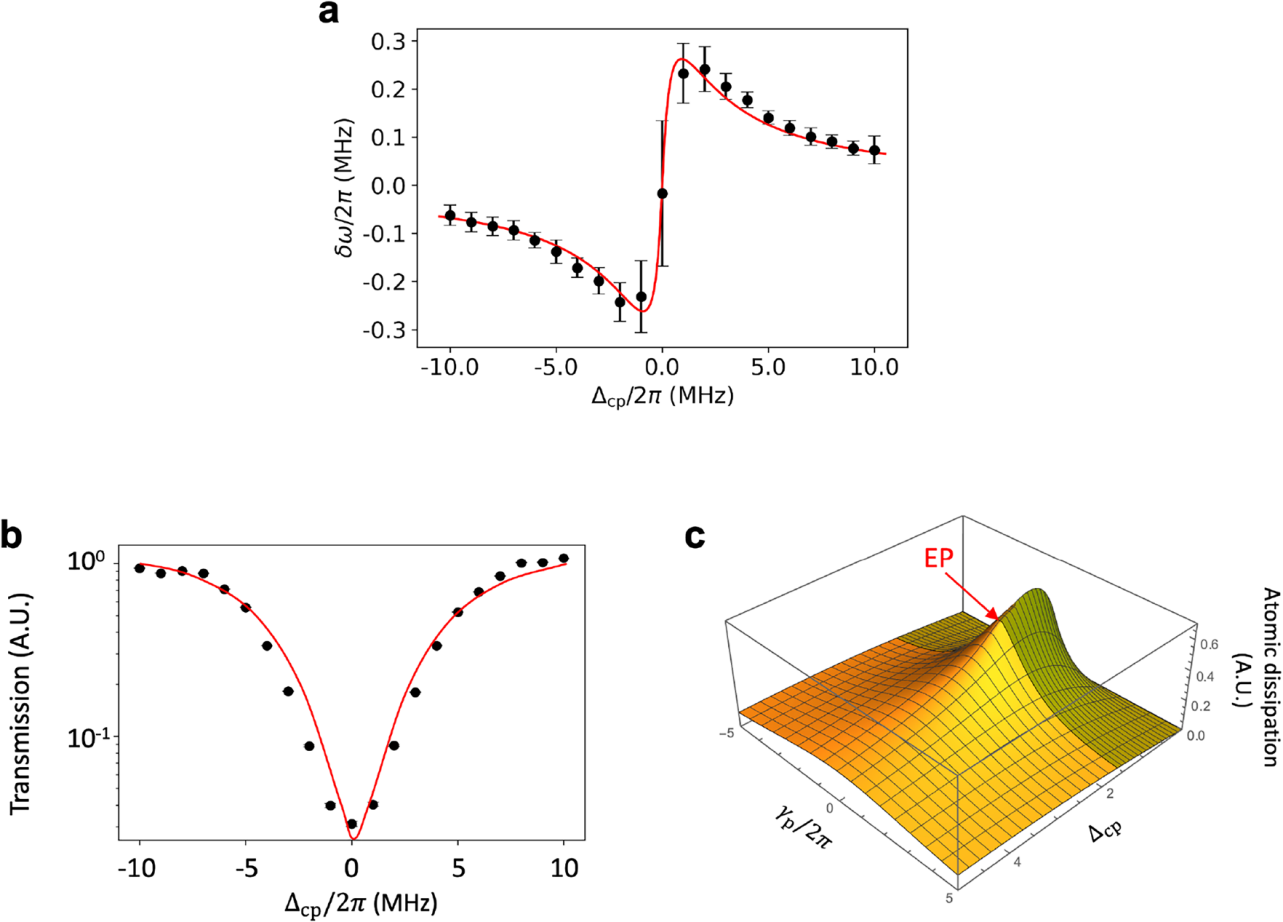
Observed frequency pushing in the atom–cavity system. (**a**) Frequency pushing values measured as a function of $\Delta _{cp}$, the cavity–atom detuning, are shown as black circles and a theoretical fit based on the semiclassical model is shown in a red solid curve with the fitting parameter $\alpha =1.20$. The other known or predetermined parameters are the same as in Fig. 3. (**b**) Peak heights (black dots) of the observed cavity transmission lineshape are shown as a function of $\Delta _{cp}$. The peak height is minimized when $\Delta _{cp}=0$, corresponding to the highest proximity to the EP under the experimental condition. A theoretical fit based on the semiclassical model is shown as red solid curve. (**c**) The power dissipated by atoms with respect to the peak power excited in the cavity-like mode calculated and plotted in the parameter space spanned by $\Delta _{cp}$ and $\gamma _p/2\pi$, both in MHz. A maximum occurs not far from the EP, at which a slope discontinuity is noted.

The frequency shift is given by $\delta \omega =\omega _t-\omega _c$, which is plotted as a function of the cavity–atom detuning, $\Delta _{cp}=\omega _c-\omega _p$ in Fig. 5a. The frequency shift is positive(negative) for a positive(negative) cavity–atom detuning, indicating the cavity resonance is shifted away from the atomic resonance, *i.e.*, frequency pushing. A theoretical fit based on Eq. (7) with a sole fitting parameter $\alpha =1.20$ is also shown along with the observation, exhibiting a good agreement between them.

## Discussion

The magnitude of frequency pushing is maximized at $\Delta _{cp}/2\pi \sim \pm 1$ MHz and the maximum frequency pushing is 41±7 kHz/per atom. This value is about 20 times larger than the maximum frequency pulling per atom observed in the cavity-QED microlaser^14^ with $N\sim 250$ and $g/2\pi \simeq 190$ kHz – well above the lasing threshold in the highly nonlinear region – generating intensity squeezed output^4^. The frequency pulling in the microlaser was influenced by many factors such as quantum jumps^28^ and photon number stabilization^4^, so a direct comparison with the frequency pushing here is difficult. But one of the reasons why the present frequency shift is larger than those in the previous studies by an order of magnitude is that the frequency shift was maximized here by choosing the experimental conditions very close to the EP, near which we can still have a single peak lineshape while getting closer to the strong atom–cavity coupling regime.

The frequency shift would increase with the atom–cavity coupling as seen in the cavity-QED frequency pulling experiment^14^. However, if we increase the coupling too much, we are in the strong coupling regime and the spectrum would have two peaks (atom–cavity mixed together) so that we cannot associate a frequency shift to the cavity resonance. We can increase the coupling up to the near-EP condition, at which the cavity transmission lineshape still has a single peak. In this way, we can greatly enhance the frequency pushing effect.

Figure 4b shows that the imaginary part $\Gamma _+$($\Gamma _-$) of the cavity-like eigenvalue for $\Delta _{cp}>0$($\Delta _{cp}<0$) is maximized at zero cavity–atom detuning, corresponding to the highest proximity to the EP condition. The difference $\Gamma _\pm - \gamma _c$ can be interpreted as the increase of the loss in the cavity-like mode due to the atom–cavity interaction. This additional loss would appear as the increased absorption of the probe laser by the atomic dissipation, and consequently the peak value of the cavity transmission becomes the lowest at the zero cavity–atom detuning as shown in Fig. 5b. In order to examine how much atomic dissipation occurs around the EP, we calculated $\gamma _p |\mathcal {P}|^2$ in the $\gamma _p$-$\Delta _{cp}$ parameter space. Fig. 5c shows this quantity divided by $|\mathcal {E}|^2$, both evaluated along the cavity-like mode, where $\mathcal {E}$ is given by Eq. (3) and $\mathcal {P}\propto \mathcal {E}/(\gamma _p-i\Delta _p)$. It quantifies the atomic dissipation normalized with respect to cavity mode excitation. A maximum is slightly shifted to the strong coupling side (shaded in Fig. 5c), but not far from the EP, at which a slope discontinuity is noted.

The fact that the maximum value is shifted toward the strong coupling regime can be regarded as an artifact. It is because the expression $\gamma _p |\mathcal {P}|^2/|\mathcal {E}|^2$ would no longer correspond to the normalized atomic dissipation in the strong coupling regime where the distinction between atom-like and the cavity-like modes becomes ambiguous. Therefore, we can reason that the atomic dissipation via the energy transfer from the cavity photons to the atoms is most efficient around the EP under the condition that the distinction between atom-like and cavity-like modes should be possible.

### Frequency pushing in the Lorentz model

One may argue that the frequency pushing can be attributed to the change of refractive index by atoms near the atomic resonance. By using the Lorentz model treating atoms as damped electron oscillators of frequency $\omega _0$ driven by an external electric field of frequency $\omega$, one can calculate the polarization density ${\textbf {P}}=Ne{\textbf {x}}/V=\chi {\textbf {E}}$ and from the real part of the electric susceptibility $\chi$, one can obtain the refractive index as $n(\omega )=\text{Re}[\sqrt{1+4\pi \chi (\omega )}]\simeq 1+2\pi \text{Re}[\chi (\omega )]$ in the Gaussian unit. The frequency shift is then given by $\delta \omega =\omega _c /n(\omega )- \omega _c \simeq \omega _c[1-n(\omega )]=-2\pi \text{Re}[\chi (\omega )]\omega _c$. Since $n <1 (n>1)$ for $\omega >\omega _0 (\omega <\omega _0)$, we obtain $\delta \omega >0 (\delta \omega <0)$, indicating frequency pushing.

However, the refractive index picture fails to provide the correct magnitude of the frequency pushing. It produces a magnitude 320 times larger than that of the observation as shown in Fig. S1 in Supplementary Note 1. It is because in the Lorentz model the polarization density is proportional to the classical radiative damping rate $\Gamma _{cl}=2e^2\omega _0^2/(3mc^3)$ whereas in quantum mechanics the polarization density is proportional to the radiative decay rate $\Gamma _{qm}=4\mu ^2\omega _0^3/(3\hbar c^3)$. For the resonance frequency $\omega _0$ of the atom, we obtain $\Gamma _{cl}/2\pi =5.65$ MHz, which should be compared with $\Gamma _{qm}/2\pi =47.6$ kHz the actual radiative decay rate of $^3$P$_1\rightarrow ^1$S$_0$ transition of atomic barium. The ratio becomes $\Gamma _{cl}/\Gamma _{qm}\approx 119$, explaining the two-orders of magnitude difference noted in Fig. S1. The remaining discrepancy might be due to the fact that in the Lorentz model, the electric field is an external field whereas in the experiment the electric field is the cavity field interacting with the atoms. So it can be modified by the atoms (e.g., the increased probe absorption via atomic dissipation), resulting in a different magnitude of frequency pushing. The details of the refractive index calculation are given in Supplementary Note 1.

In summary, we have measured the frequency pushing of the cavity resonance in a setting of the cavity-QED microlaser with the injected $^{138}$Ba atoms initially prepared in the ground state. The observed frequency pushing data were analyzed with the semiclassical Maxwell–Schrödinger equation. The cavity transmission lineshape derived from the theory fit the data well when the transit time broadening of the passing atoms in the presence of a probe laser was incorporated in the dephasing rate of the atomic-induced dipole moment. The amount of the maximum frequency pushing was $41\pm 7$ kHz per atom with about 6 atoms in the cavity on average. The experimental parameters under which our observation is performed correspond to the intermediate coupling regime of cavity QED, close to the condition for an exceptional point (EP) in the atom–cavity composite. The frequency pushing was enhanced by the high proximity to the EP, which is confirmed by the real and imaginary parts of the eigenfrequencies extracted from the observed cavity transmission lineshapes. The probe absorption due to the energy transfer from the cavity photons to the atoms was most efficient at zero cavity–atom detuning due to the high proximity to the EP.

The frequency shift of the cavity resonance is shown to have a dependence on the atomic coherence of initial atomic states such as in quantum superposition states. Experimental verification of such coherence-induced frequency shifts would be interesting as an extension of the present study.

## Methods

### Derivation of Eqs. (1) and (2)

We start with the wave equation for the electric field **E** obtained from the source-free Maxwell equations. In the presence of an induced polarization density **P** of an atom by the electric field, the wave equation can be written as^29^

16 $$\begin{aligned} \nabla ^2{\textbf {E}}-\frac{1}{c^2}\frac{\partial ^2{\textbf {E}}}{\partial {t}^2} = \frac{4{\pi }}{c^2}\frac{\partial ^2 {\textbf {P}}}{\partial t^2}-4\pi \nabla (\nabla \cdot {{\textbf {P}}}). \end{aligned}$$

Let us assume **E** is the electric field in a cavity and both $\mathrm{\textbf{E}}$ and $\mathrm{\textbf{P}}$ are polarized in a particular direction (*x* direction) having sinusoidal variation along *z* direction (so $\nabla \cdot {{\textbf {P}}}\simeq 0$). We can then rewrite the equation in terms of the cavity-field amplitudes $E_c$ evaluated at the location of the atom and the corresponding atomic polarization *P* as

17 $$\begin{aligned} \ddot{E}_c (t)+{\omega }_c ^2 E_c (t) = -4{\pi } \ddot{P}(t) \end{aligned}$$

where $\omega _c$ is the frequency of the cavity field.

From the Schrödinger equation or equivalently from the equation for the density matrix $\rho$ with the interaction Hamiltonian $H_I=-\mu E_c \cos \omega _c t$, we can obtain the equation for the induced dipole moment $p(\equiv \mu \rho _{ab}+\mathrm{h.c.})$ for a single atom, where $\mu \equiv \langle \text{a} | ex | \text{b}\rangle$ and the upper and lower levels are denoted as a and b, respectively. The resulting equation^30^ is

18 $$\begin{aligned} \ddot{p}+2\gamma _p p+\omega _p ^2 p=-\frac{2\mu ^2}{\hbar }\omega _p E_c \sigma , \end{aligned}$$

where $\gamma _p$ is the damping rate (HWHM) of the induced dipole, $\sigma (\equiv \rho _{aa}-\rho _{bb})$ is the population inversion and $\hbar \omega _p=\hbar (\omega _a-\omega _b)$ is the energy difference between levels a and b.

In the presence of a probe laser field $E_L$ with a probe-cavity coupling $\xi$ (in the unit of frequency squared), the equation for the cavity field at the position of the atom can be rewritten as^30^

19 $$\begin{aligned} \ddot{E}_c+2\gamma _c E_c+\omega _c^2 E_c\simeq \frac{4\pi \omega _p^2}{V}p+\xi E_L \end{aligned}$$

where $\gamma _c$ is the cavity decay rate (HWHM) newly introduced symmetrically with respect to the atomic decay rate in Eq. (18). In addition, the polarization density *P* is replaced with *p*/*V* with *V* the cavity mode volume and we use the approximation $\ddot{P}\simeq -\omega _p^2 P$.

We can further simplify the coupled Maxwell–Schrödinger equations, Eqs. (18) and (19), by using the slowly varying envelope approximation with $E_c(t)={\text {Re}}[\mathcal {E}(t)e^{-i\omega t}]$, $\frac{p(t)}{V}={\text {Re}}[\mathcal {P}(t)e^{-i\omega t}]$ and $E_L=E_0\cos {\omega t}$. For *N* ground state atoms, $\sigma$ is replaced with $(-N)$. The number *N* of atoms is defined as $N=\sum _{i} |\psi (\mathbf{\mathrm r}_i)|^2$ with $\psi (\mathbf{\mathrm r}_i)$ is the mode function evaluated at the location of *i*th atom. With $\Delta _{p(c)}=\omega -\omega _{p(c)}$, the coupled Maxwell–Schrödinger equations are reduced to Eqs. (1) and (2). Similar treatments can be found in the literature^24,30^.

### Experimental setup and data calibration method

**Figure 6:**
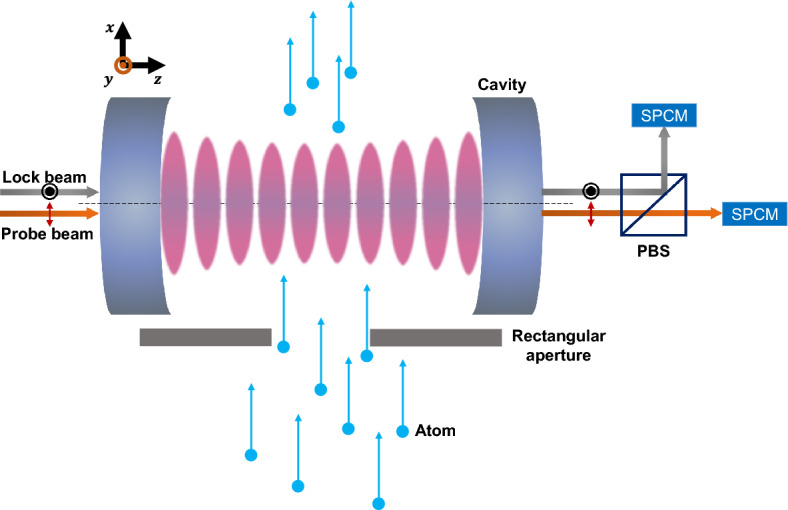
Top view of the experimental setup. The rectangular aperture is located in the atomic beam path, and a probe laser and a cavity-locking laser are injected along the cavity axis. The transmission of two lasers with perpendicular polarization is separated by a PBS and measured with SPCMs.

The experimental setup is depicted in Fig. 6. The ground state $^{138}$Ba atoms in a collimated beam, ejected from an oven driven by current via Joule heating, go through a TEM$_{00}$ mode of a Fabry-Pèrot cavity perpendicularly to the cavity axis at a mean velocity $v=630$ m/s. The cavity made of two identical mirrors of a 10-cm radius of curvature has a length of 1.0 mm and thus the mode waist $w_m$ of the TEM$_{00}$ mode is 42 $\upmu \text{m}$, resulting in a transit time $\tau =\sqrt{\pi }w_m/v=120$ ns. The finesse of the cavity is 1.0 million, resulting in a cavity decay rate of $\gamma _c/2\pi =75$ kHz. The cavity resonance is tuned to the $^1$S$_0\leftrightarrow ^3$P$_1$ transition of barium at a wavelength of 791 nm. The upper $^3$P$_1$ level has a total decay rate of $\Gamma _0/2\pi =117.8$ kHz: it decays to $^1$S$_0$ at a rate of 47.6 kHz, to $^3$D$_2$ state at 50.6 kHz and to $^3$D$_1$ state at 19.6 kHz^31^.

A rectangular atomic-beam aperture of a width of 250 $\upmu \text{m}$ and a height of 25 $\upmu \text{m}$ is placed in front of the cavity mode in order to confine the atomic beam in the cavity mode in the transverse directions. The coupling constant associated with the standing-wave cavity mode is given by $g/2\pi =348$ kHz.

Due to the birefringence coming from the particular shape of the cavity mirrors^32^, the resonance frequency of the TEM$_{00}$ mode has strong polarization dependence. The resonance frequencies for the horizontal (in *x* direction in Fig. 6) and vertical (in *y* direction) polarizations are separated by about 4 MHz. The vertical polarization is used for a cavity locking laser and the horizontal polarization is used for a probe laser for the frequency shift measurements^10,15,16^. Both lasers are independently scanned using separate acousto-optic modulators (AOMs) of a scan range of about 20 MHz. A polarizing beam splitter with a high extinction ratio is used to separate the laser beams with different polarizations behind the cavity. The cavity resonance is detuned from the atomic resonance in a range from − 10 MHz to + 10 MHz by changing the detuning of the cavity locking laser accordingly. For a given cavity–atom detuning, the probe laser is scanned across the (modified) cavity resonance, and the cavity transmission is measured with a single-photon counting module (SPCM) to obtain the cavity transmission lineshape as in Fig. 3. The mean number of probe photons in the cavity is much less than one per atom throughout the measurements to satisfy the condition that atoms are mostly in the ground state.

The mean number *N* of atoms in the cavity are calibrated using the technique of n-vs-N curve of the cavity-QED microlaser^4,10,15,16^. In a nutshell, the unique characteristics in the lasing of the atom–cavity system are utilized. The atom–cavity system is operated as a microlaser with the injected atoms initially in the excited state. The mean number *n* of photons in the cavity is then measured as a function of the mean number *N* of atoms while the fluorescence from $^1$P$_1$ to $^1$S$_0$ transition (at 553 nm) of the atoms proportional to the atomic beam flux is measured simultaneously. The resulting n-vs-N curve exhibits quantum jumps at unique sets of *n* and *N*^28^, predicted by the quantum microlaser theory^33^. Comparing the n-v-N data and the fluorescence data, one can calibrate *N* values for a given atomic beam flux. By using this technique, the mean number of atoms in the cavity is obtained to be $N=5.8$ for the frequency pushing measurements.

The most probable velocity *v* of atoms is obtained from the Doppler shift. By employing a counter-propagating probe laser, scanning it across the $^1$S$_0 \leftrightarrow ^1$P$_1$ transition at $\lambda =553$ nm and measuring the fluorescence, we obtain a velocity distribution curve reflecting the Doppler shifts of various velocity components. The peak of the distribution corresponding to the most probable velocity *v* is shifted from the zero-velocity resonance by $v/\lambda$ in Hz. The zero-velocity resonance is measured by a separate probe laser intersecting the atomic beam perpendicularly.

The velocity of atoms is controlled by the current going through the oven made of tantalum tubing. By changing the oven current from 240 A to 360 A, the velocity of atoms can be changed from 540 m/s to 740 m/s. For the present experiment, we specifically chose $v=630$ m/s to make the atomic dephasing rate $\gamma _{\text{p}}$ proportional to the velocity satisfy the EP condition.

### Supplementary Information

Supplementary Information.

## Data Availability

The datasets generated during the current study are available from the corresponding author on reasonable request.

## Code availability

The code that supports the findings of this study are available from the corresponding author upon reasonable request.
