# Supplementary material for: Frequency pushing enhanced by an exceptional point in an atom–cavity coupled system
Source: Sci Rep. 2024 Feb 12;14:3471. doi: 10.1038/s41598-024-54008-w (PMC11306339; doi:10.1038/s41598-024-54008-w)
Supplement: Supplementary file 1 — Supplementary Information. [file 41598_2024_54008_MOESM1_ESM.pdf]

Supplementary Materials

1. Refractive Index Approach Based on the Lorentz Model

A medium made of atoms with one valence electron can be classically described in terms of electron damped harmonic oscillators. We assume that an electron is bound to a restoring force of oscillation frequency of ω_0 and subject to a damping force proportional to its velocity with a damping rate Γ. The equation of motion for an electron's displacement x is basically that of a damped harmonic oscillator. When it is driven by an external electric field $E(t) = E_0 e^{-i\omega t}$, the equation of motion can be written as

$$\frac{d^2x}{dt^2} + \Gamma\frac{dx}{dt} + \omega_0^2 x = \frac{eE_0 e^{-i\omega t}}{m}, \tag{S1}$$

where $e(< 0)$ and m are the charge and the mass of an electron, respectively. The steady-state solution near resonance is

$$x(t) = -\frac{(eE_0/m)e^{-i\omega t}}{(\omega^2 - \omega_0^2) + i\Gamma\omega} \simeq -\frac{(eE/2m\omega_0)}{(\omega - \omega_0) + i\Gamma/2}. \tag{S2}$$

The polarization density is given by $P = Nex/V = \chi E$ with N/V the density of the oscillators and χ the electric susceptibility. The dielectric constant in the Gaussian unit is given by

$$\epsilon(\omega) = 1 + 4\pi\chi(\omega) = 1 - 4\pi\frac{Ne^2/(2m\omega V)}{(\omega - \omega_0) + i\Gamma/2}, \tag{S3}$$

and the real part of the refractive index is

$$n_{\rm r}(\omega) = \mathrm{Re}\sqrt{\epsilon(\omega)} \simeq 1 - \frac{\pi Ne^2}{m\omega V}\frac{\omega - \omega_0}{(\omega - \omega_0)^2 + (\Gamma/2)^2}. \tag{S4}$$

In the presence of a medium of refractive index $n > 1$, the optical path length associated with the cavity length is increased, making the resonance wavelength larger or the resonance frequency be changed from $\omega_{\rm c}$ to $\omega_{\rm c}/n$, resulting in a shift of cavity resonance, $\delta\omega = \omega_{\rm c}/n - \omega_{\rm c}$. For $n \sim 1$, $\delta\omega \simeq \omega_{\rm c}(1-n) \simeq \frac{\pi Ne^2}{mV}\frac{\omega-\omega_0}{(\omega-\omega_0)^2+(\Gamma/2)^2}$, clearly showing the cavity resonance is pushed away from the oscillator resonance, *i.e.*, frequency pushing.

The maximum frequency pushing occurs when $\omega - \omega_0 = \Gamma/2$, which is different from the semiclassical theory where the maximum frequency shift moves toward the zero detuning because of the atom-cavity coupling. In addition, when we evaluate the maximum frequency pushing, $\delta\omega_{\rm max} = \frac{\pi Ne^2}{mV\Gamma}$, with $N = 5.8$, V equal to the cavity mode volume($= 4.5 \times 10^{-13}\mathrm{m}^3$)

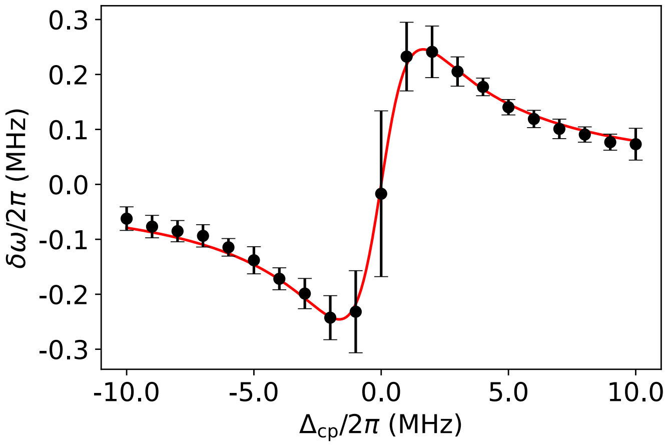

FIG. S1. **Fitting the observed frequency pushing with the Lorentz model.** Frequency pushing of the cavity field due to the near-resonant refractive index according to the Lorentz model. It has been scaled down by a factor of 1/320 in order to fit the data. Parameters are $N = 5.8$, $\Gamma = 2\pi \times 3.3$ MHz corresponding to $2\gamma_{\rm p}$ in the semiclassical model.

and $\Gamma/2\pi = 3.3$ MHz, we obtain $\delta\omega_{\rm max}/2\pi = 76.8$ MHz, which is 320 times larger than the observed maximum of 0.24 MHz in Fig. S1.

This two-orders-of-magnitude difference can be associated with the difference in the magnitudes of the classical polarization density $|P| = |Nex/V|_{\rm max}$ obtainable from Eq. (S2) and that of the semiclassical polarization density $|\mathcal{P}|_{\rm max}$ in Eqs. (2) and (3). The maximum of the classical polarization occurs when $\omega - \omega_0 = \Gamma/2$ and its value is

$$|P|_{\rm max} = \frac{(N/V)e^2 E_0}{\sqrt{2} m\omega_0 \Gamma}. \tag{S5}$$

On the other hand, from Eq. (2), we obtain

$$\mathcal{P}(\omega) = i\frac{\mu^2 N\mathcal{E}}{\hbar V}\frac{1}{\gamma_{\rm p} - i\Delta_{\rm p}} \tag{S6}$$

in the steady state. The maximum magnitude is estimated with $\Delta_{\rm c} \sim \Delta_{\rm p} \sim \gamma_{\rm p}$,

$$|\mathcal{P}|_{\rm max} \sim \frac{(N/V)\mu^2|\mathcal{E}|_{\rm max}}{\sqrt{2}\hbar\gamma_{\rm p}}. \tag{S7}$$

Treating $|\mathcal{E}|_{\rm max}$ and E_0 equivalently and $\Gamma = 2\gamma_{\rm p}$, we then obtain the ratio as

$$\frac{|P|_{\rm max}}{|\mathcal{P}|_{\rm max}} \sim \frac{e^2\hbar}{2m\omega_0\mu^2}. \tag{S8}$$

This ratio is exactly the ratio of the radiative damping rate $\Gamma_{\rm cl}$ in classical electromagnetism to the radiative decay rate $\Gamma_{\rm qm}$ in quantum mechanics. In the Gaussian unit,

$$\Gamma_{\rm cl} = \frac{2e^2\omega_0^2}{3mc^3}, \quad \Gamma_{\rm qm} = \frac{4\mu^2\omega_0^3}{3\hbar c^3}, \tag{S9}$$

and the ratio is

$$\frac{\Gamma_{\rm cl}}{\Gamma_{\rm qm}} = \frac{e^2\hbar}{2m\omega_0\mu^2}, \tag{S10}$$

the same as $|P|_{\rm max}/|\mathcal{P}|_{\rm max}$. The value of $\Gamma_{\rm cl}$ is evaluated to be $2\pi \times 5.62$ MHz whereas the value of $\Gamma_{\rm qm}$ or the radiative decay rate of $^3\mathrm{P}_1 \rightarrow {}^1\mathrm{S}_0$ transition of atomic barium is $2\pi \times 47.6$ kHz[26], so $\Gamma_{\rm cl}/\Gamma_{\rm qm} \simeq 119$, explaining the aforementioned two-orders-of-magnitude difference.
